# Safety, Efficacy, and Pharmacokinetics of Unecritinib (TQ‐B3101) in Pediatric Patients With Relapsed/Refractory Anaplastic Lymphoma Kinase‐Positive Anaplastic Large‐Cell Lymphoma: A Phase I Study

**DOI:** 10.1002/mco2.71128

**Published:** 2026-11-05

**Authors:** Suying Lu, Junting Huang, Ruiqing Cai, Yan Gao, Jia Zhu, Juan Wang, Yi Que, Zijun Zhen, Feifei Sun, Huiqiang Huang, Yizhuo Zhang

**Affiliations:** ^1^ Department of Pediatric Oncology State Key Laboratory of Oncology in South China Guangdong Provincial Clinical Research Center For Cancer Collaborative Innovation Center For Cancer Medicine Sun Yat‐sen University Cancer Center Guangzhou People's Republic of China; ^2^ Department of Medical Oncology State Key Laboratory of Oncology in South China Guangdong Provincial Clinical Research Center For Cancer Collaborative Innovation Center For Cancer Medicine Sun Yat‐sen University Cancer Center Guangzhou People's Republic of China

**Keywords:** anaplastic large‐cell lymphoma, anaplastic lymphoma kinase‐positive, relapsed/refractory, unecritinib

## Abstract

This study evaluated the safety, pharmacokinetics (PK), and efficacy of unecritinib, a novel ROS1/anaplastic lymphoma kinase (ALK)/c‐MET inhibitor, in pediatric patients with relapsed/refractory (R/R) ALK‐positive (ALK+) anaplastic large‐cell lymphoma (ALCL). This was an open‐label, single‐arm, Phase I study using a 3+3 dose‐escalation design in patients aged 10–18 years. The primary endpoints were safety, determination of the recommended phase II dose (RP2D), and PK profiling. Secondary endpoints included overall response rate (ORR), progression‐free survival (PFS), and overall survival (OS). Nine patients were evaluable for toxicity and efficacy analyses. No dose‐limiting toxicities were observed. The most frequent treatment‐related adverse events were neutropenia, leukopenia, vomiting, and nausea, predominantly Grade 1–2. PK analysis showed rapid metabolism of unecritinib by carboxylesterase to its active metabolite unecritinib M, with a *T*_max_ of approximately 3 h. The ORR was 100% (six complete and three partial responses). The 3‐year PFS and OS rates were 53.3% and 100%, respectively. Genomic alterations in *TP53*, *MYC*, *KMT2D*, and *MYCL1* were identified in a subset of patients, suggesting potential prognostic heterogeneity. Unecritinib demonstrated a favorable safety profile and promising preliminary efficacy in patients with R/R ALK+ ALCL. The RP2D was 250 mg twice daily for patients aged > 10 years.

**Trial Registration**: ClinicalTrials.gov identifier: NCT04306887

## Graphical abstract

This Phase I study evaluated unecritinib (TQ‐B3101) in pediatric patients with relapsed/refractory ALK+ anaplastic large‐cell lymphoma. Unecritinib showed a manageable safety profile without dose‐limiting toxicities and achieved remarkable efficacy, with a 100% overall response rate. With favorable pharmacokinetics and 100% 3‐year overall survival, unecritinib represents a promising and potent therapeutic strategy for this challenging pediatric population.

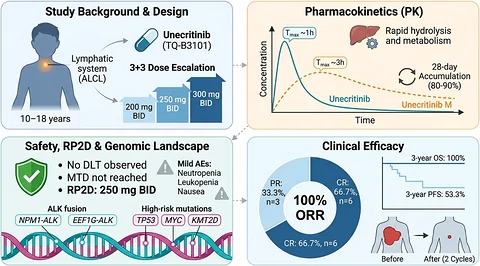

## 1 Introduction

The anaplastic lymphoma kinase (ALK) oncogene encodes a receptor tyrosine kinase that can undergo oncogenic activation through translocation, point mutations, or gene amplification [1]. Anaplastic large‐cell lymphoma (ALCL) is a well‐recognized and distinct subtype of T‐cell non‐Hodgkin lymphoma that accounts for 10%–15% of all childhood lymphomas [2, 3, 4]. Most pediatric patients with ALCL harbor characteristic ALK gene translocations accompanied by ALK protein overexpression. The World Health Organization categorizes ALCL into two distinct subgroups according to chromosomal translocations involving the *ALK* gene: ALK‐positive (ALK+) ALCL and ALK‐negative (ALK−) ALCL [5]. More than 92% of pediatric patients with ALCL have ALK+ disease [6]. Despite differences in therapeutic agents, treatment intensity, and treatment duration across previous clinical trials for ALCL [7, 8, 9, 10, 11], approximately 30% of patients continue to experience failure of first‐line therapy.

Unecritinib (TQ‐B3101) is a novel inhibitor of ROS1, ALK, and c‐MET kinases [12, 13] derived from crizotinib through structural modification of the pyridine ring. Unecritinib was approved by the National Medical Products Administration for adult patients with ALK+ non‐small‐cell lung cancer (NSCLC) in China and was launched in China on April 30, 2024. It is a highly potent, orally active, blood–brain barrier‐permeable inhibitor with relatively low toxicity and demonstrated antitumor activity [13]. These properties suggest potential utility in the treatment of pediatric patients with ALK+ ALCL. Preclinical studies showed that unecritinib is rapidly and extensively converted to its active metabolite (M), unecritinib M, through N‐deacetylation [13]. In animal models, unecritinib markedly inhibited phosphorylation of AKT and extracellular signal‐regulated kinase 1/2 within the downstream ALK signaling pathway in tumor tissues [13]. Compared with equimolar crizotinib, unecritinib demonstrated longer‐lasting and more potent inhibitory activity [13]. However, the safety and efficacy of unecritinib in pediatric patients with cancer remain unknown.

Previous studies, including ADVL0912 [14] and A8081013 [15], demonstrated favorable efficacy and safety of the ALK inhibitor crizotinib in the treatment of ALK+ ALCL. Crizotinib has also been combined with standard chemotherapy regimens, such as ALCL99, to reduce the risk of relapse [8]. Ceritinib [16] and alectinib [17] have likewise demonstrated promising preliminary antitumor activity in patients with relapsed/refractory (R/R) ALK+ ALCL.

Despite increasing evidence supporting the use of ALK inhibitors in pediatric patients, clinical data regarding domestically developed ALK inhibitors remain limited. Moreover, although survival outcomes have improved substantially among pediatric patients with ALK+ ALCL treated with crizotinib, treatment‐related adverse events (TRAEs), including ocular and gastrointestinal toxicities, remain clinically important concerns [18, 19]. Preclinical studies demonstrated that unecritinib may carry a lower risk of ocular toxicity [18, 19] because unecritinib M showed approximately 200‐fold lower distribution in ocular tissues than crizotinib [13]. These findings support further investigation of unecritinib as a potential therapeutic option for pediatric patients with ALK+ ALCL.

Data regarding the efficacy of ALK inhibitors in pediatric patients in China remain limited despite substantial unmet clinical need in this population. In this study, we evaluated the safety and tolerability of unecritinib in pediatric patients with R/R ALK+ ALCL. We also assessed its pharmacokinetics (PK) and explored preliminary antitumor activity. In addition, we characterized the genomic landscape of pediatric patients treated with unecritinib to explore potential molecular features associated with prognosis. To our knowledge, this study represents the first prospective clinical investigation of unecritinib in pediatric patients. Unecritinib demonstrated a favorable safety profile, with no dose‐limiting toxicities (DLTs) observed, and objective responses were achieved in all treated patients. These findings provide preliminary evidence supporting the potential of unecritinib as a therapeutic option for pediatric patients with ALK+ ALCL and establish a foundation for future clinical development.

## 2 Results

### 2.1 Patient Characteristics

Between November 29, 2019, and December 30, 2021, nine pediatric patients with predominantly R/R ALK+ ALCL were screened, enrolled, and included in the efficacy and safety analyses. Table 1 summarizes baseline demographic and disease‐related characteristics of the enrolled cohort, which were used to contextualize interpretation of safety and treatment outcomes. Among the enrolled patients, six had relapsed disease, two had progressive disease (PD), and one had refractory disease. At the data cutoff date of May 31, 2024, the median follow‐up duration was 52.1 months (range, 29.4–54.8).

**TABLE 1:** Baseline characteristics.

Case no.	Age, y		Sex	Height, cm	Weight, kg	Histological subtype	Stage at diagnosis^a^	Previous lines of chemotherapy^b^	Time from treatment discontinuation to relapse/progression	Disease sites at enrollment	Method used to assess ALK abnormalities	Disease status at enrollment	ECOG PS
P1	14	M		161.0	41.5	Common pattern	III	1	8 months	Lymph nodes, bone	FISH	Relapsed	0
P2	11	M		146.0	42.0	Common pattern	III	1	20 months	Lymph nodes	IHC	Relapsed	0
P3	12	F		152.0	37.5	Common pattern	III	1	Residual disease after first‐line chemotherapy	Lymph nodes, soft tissue	IHC	Refractory	0
P4	14	M		164.0	68.0	Small‐cell pattern	III	2^c^	Relapse during maintenance vinblastine therapy after achieving CR	Lymph nodes	IHC	Relapsed	0
P5	12	F		143.5	32.9	Common pattern	III	1	3 months	Lymph nodes, lung, soft tissue	IHC	Relapsed	0
P6	14	M		169.5	54.1	Small‐cell pattern	III	1	14 months	Lymph nodes	IHC	Relapsed	0
P7	10	F		143.5	33.8	Common pattern	III	1	1.5 months	Lymph nodes, lung, bone, pericardium, pleura, bronchus, pancreas	IHC	Progressive	1
P8	16	M		167.0	52.0	Common pattern	IV^d^	1	Disease progression after 4 cycles of chemotherapy	Bone	IHC	Progressive	0
P9	10	F		129.0	26.1	Lymphohistiocytic pattern	IV^e^	1	1 month	Skull base, lymph nodes, bone, lung, pancreas, abdominal cavity, skin, muscle	IHC	Relapsed	0

Abbreviations: ALK, anaplastic lymphoma kinase; CR, complete response; ECOG PS, Eastern Cooperative Oncology Group performance status; F, female; FISH, fluorescence in situ hybridization; IHC, immunohistochemistry; M, male; no., number; P, patient; y, year.

^a^ Staging was performed according to the Revised International Pediatric Non‐Hodgkin Lymphoma Staging System [20].

^b^ All patients received the Non‐Hodgkin Lymphoma Berlin–Frankfurt–Münster 1990 (NHL‐BFM‐90) regimen as first‐line chemotherapy [21].

^c^ The second‐line regimen administered to Patient 4 was vinblastine.

^d^ Stage IV disease was assigned because of bone marrow involvement.

^e^ Stage IV disease was assigned because of central nervous system involvement.

### 2.2 Safety, Maximum Tolerated Dose, and Recommended Phase II Dose

Table 2 summarizes TRAEs observed during unecritinib administration across dose levels of 200, 250, and 300 mg twice daily (BID). The safety evaluation aimed to characterize toxicity patterns, identify DLTs, and determine the maximum tolerated dose (MTD) and recommended phase II dose (RP2D) in pediatric patients with R/R ALK+ ALCL. Safety grading was performed using CTCAE v5.0 across all treated patients.

**TABLE 2:** Treatment‐related adverse events.

	Overall		200 mg BID		250 mg BID		300 mg BID	
	(*n* = 9)		(*n* = 3)		(*n* = 3)		(*n* = 3)	
	Any grade,	Grade 3,	Any grade,	Grade 3,	Any grade,	Grade 3,	Any grade,	Grade 3,
	*n* (%)	*n* (%)	*n* (%)	*n* (%)	*n* (%)	*n* (%)	*n* (%)	*n* (%)
**Hematologic toxicities**								
Neutropenia	8 (88.9)	6 (66.7)	2 (66.7)	2 (66.7)	3 (100)	1 (33.3)	3 (100)	3 (100)
Leukopenia	7 (77.8)	2 (22.2)	2 (66.7)	2 (66.7)	2 (66.7)	0	3 (100)	0
Decreased lymphocyte count	3 (33.3)	0	2 (66.7)	0	1 (33.3)	0	0	0
Anemia	2 (22.2)	0	1 (33.3)	0	0	0	1 (33.3)	0
Thrombocytopenia	1 (11.1)	0	1 (33.3)	0	0	0	0	0
**Nonhematologic toxicities**								
Vomiting	7 (77.8)	0	3 (100)	0	2 (66.7)	0	2 (66.7)	0
Nausea	7 (77.8)	0	3 (100)	0	3 (100)	0	1 (33.3)	0
Alanine aminotransferase increased	6 (66.7)	0	2 (66.7)	0	2 (66.7)	0	2 (66.7)	0
Aspartate aminotransferase increased	6 (66.7)	1 (11.1)	2 (66.7)	1 (33.3)	1 (33.3)	0	3 (100)	0
Rash	6 (66.7)	0	2 (66.7)	0	2 (66.7)	0	2 (66.7)	0
Proteinuria	5 (55.6)	0	1 (33.3)	0	2 (66.7)	0	2 (66.7)	0
Diarrhea	5 (55.6)	0	1 (33.3)	0	2 (66.7)	0	2 (66.7)	0
Anorexia	5 (55.6)	0	2 (66.7)	0	2 (66.7)	0	1 (33.3)	0
Sinus bradycardia	5 (55.6)	0	1 (33.3)	0	2 (66.7)	0	2 (66.7)	0
Abdominal pain	4 (44.4)	1 (11.1)	0	0	3 (100)	0	1 (33.3)	1 (33.3)
Hypotension	3 (33.3)	0	1 (33.3)	0	2 (66.7)	0	0	0
Sinus tachycardia	3 (33.3)	0	1 (33.3)	0	1 (33.3)	0	1 (33.3)	0
Weight gain	3 (33.3)	0	0	0	1 (33.3)	0	2 (66.7)	0
Hypoalbuminemia	3 (33.3)	1 (11.1)	1 (33.3)	0	1 (33.3)	0	1 (33.3)	1 (33.3)
Gamma‐glutamyltransferase increased	2 (22.2)	0	0	0	0	0	2 (66.7)	0
ALP increased	2 (22.2)	0	2 (66.7)	0	0	0	0	0
Blurred vision	2 (22.2)	0	0	0	1 (33.3)	0	1 (33.3)	0
Eye pain	2 (22.2)	0	0	0	0	0	2 (66.7)	0
Dizziness	2 (22.2)	0	0	0	1 (33.3)	0	1 (33.3)	0
Triglycerides increased	2 (22.2)	1 (11.1)	1 (33.3)	0	1 (33.3)	1 (33.3)	0	0
Pulmonary infection	1 (11.1)	1 (11.1)	0	0	1 (33.3)	1 (33.3)	0	0
Constipation	1 (11.1)	0	0	0	1 (33.3)	0	0	0
Intestinal obstruction	1 (11.1)	0	0	0	0	0	1 (33.3)	0
Abdominal distension	1 (11.1)	0	0	0	1 (33.3)	0	0	0
Cholesterol elevation	1 (11.1)	0	0	0	1 (33.3)	0	0	0
Creatine phosphokinase‐MB increased	1 (11.1)	0	0	0	0	0	1 (33.3)	0
Lactate dehydrogenase increased	1 (11.1)	0	1 (33.3)	0	0	0	0	0
Optic nerve damage	1 (11.1)	0	0	0	1 (33.3)	0	0	0
Weight loss	1 (11.1)	0	1 (33.3)	0	0	0	0	0
Hematuria	1 (11.1)	0	1 (33.3)	0	0	0	0	0
Hyperhidrosis	1 (11.1)	0	1 (33.3)	0	0	0	0	0
Diplopia	1 (11.1)	0	0	0	0	0	1 (33.3)	0

*Note*: Adverse events were graded according to the National Cancer Institute Common Terminology Criteria for Adverse Events (NCI‐CTCAE), version 5.0. No grade 4 or 5 treatment‐related adverse events were observed in this study.

Abbreviations: ALP, alkaline phosphatase; BID, twice daily; MB, myocardial band; *n*, number of patients.

Neutropenia was the most frequent TRAE, occurring in eight patients (88.9%) including Grade 3 events in six patients and Grade 1–2 events in two patients. Leukopenia occurred in seven patients (77.8%), consisting of five Grade 1–2 and two Grade 3 events. Vomiting and nausea were each reported in seven patients (77.8%), all Grade 1–2 in severity.

Transient Grade 3 neutropenia occurred across all evaluated dose levels. Additional Grade 3 TRAEs included increased aspartate aminotransferase (AST), abdominal pain, hypoalbuminemia, triglycerides increased, and pulmonary infection, each observed in one patient (11.1%). All Grade 3 events resolved without dose interruption or reduction. No Grade 4 TRAEs, DLTs, or treatment‐related deaths were observed. No patients discontinued therapy due to toxicity.

The most frequent nonhematologic TRAEs were Grade 1–2 gastrointestinal and biochemical abnormalities, including vomiting (77.8%), nausea (77.8%), increased alanine aminotransferase (ALT) (66.7%), increased AST (66.7%), and rash (66.7%). No cumulative toxicity or dose‐dependent long‐term safety signals were observed during follow‐up.

At data cutoff, six patients had received unecritinib for more than two years without undergoing allogeneic hematopoietic stem cell transplantation (HSCT). Long‐term follow‐up showed no delayed or late‐onset toxicities, including growth impairment or new‐onset cardiac adverse events (AEs).

No DLTs were observed at any dose level (200, 250, or 300 mg BID), and therefore the MTD was not reached. Previous adult studies demonstrated that 350 mg BID was associated with higher rates of Grade ≥ 3 TRAEs and more frequent treatment interruption or dose reduction compared with 300 mg BID [13], which established 300 mg BID as the adult RP2D.

PK comparisons showed higher exposure to unecritinib and unecritinib M in pediatric patients compared with adults at the same dose [13]; however, this did not translate into increased toxicity. Considering limited sample size, interindividual variability, body weight distribution, adult safety data, and a relatively flat exposure–efficacy relationship between 200 and 300 mg BID, 250 mg BID was selected as the RP2D for pediatric patients older than 10 years.

### 2.3 Pharmacokinetic Profile

Of the nine enrolled patients, seven underwent PK sampling, while two declined blood collection. PK analysis was therefore performed in the 200 mg (*n* = 3), 250 mg (*n* = 3), and 300 mg (*n* = 1) dose groups. The purpose of this analysis was to characterize the plasma concentration–time profile and metabolic conversion of unecritinib and its active metabolite unecritinib M in pediatric patients.

Following oral administration, unecritinib was rapidly absorbed, reaching peak plasma concentrations approximately 1 h post‐dose (Figure 1A,B). Corresponding metabolite profiles (Figure 1C,D) showed that unecritinib M reached peak concentrations (*T*_max_) at approximately 3 h post‐dose. The concentration–time curves across all dose groups demonstrated rapid absorption followed by a steep elimination phase.

**FIGURE 1:**
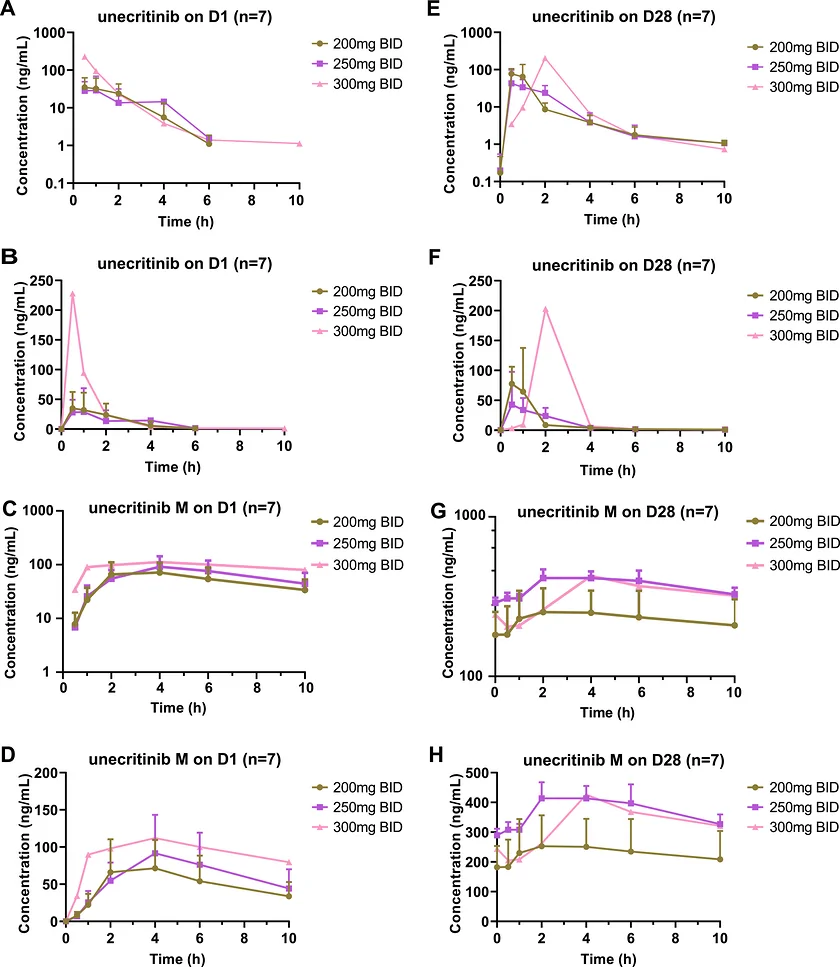
Pharmacokinetic characteristics of unecritinib and its active metabolite. (A) Logarithmic‐scale plasma concentration–time curves of unecritinib following oral administration of 200, 250, or 300 mg BID on Day 1. (B) Linear‐scale plasma concentration–time curves of unecritinib on Day 1. (C) Logarithmic‐scale plasma concentration–time curves of unecritinib M (metabolite) following oral administration of 200, 250, or 300 mg BID on Day 1. (D) Linear‐scale plasma concentration–time curves of unecritinib M on Day 1. (E) Logarithmic‐scale plasma concentration–time curves of unecritinib on Day 28. (F) Linear‐scale plasma concentration–time curves of unecritinib on Day 28. (G) Logarithmic‐scale plasma concentration–time curves of unecritinib M on Day 28. (H) Linear‐scale plasma concentration–time curves of unecritinib M on Day 28. Plasma concentrations were determined using high‐performance liquid chromatography–tandem mass spectrometry. BID, twice daily; *h*, hour; M, metabolite; *n*, number of patients.

Using intensive sampling and a validated assay with a lower limit of quantification below 1/32 of the maximum plasma concentration (*C*_max_), full PK profiles were obtained. Plasma concentrations of unecritinib declined to below 1/32 of *C*_max_ within 10 h post‐dose.

Noncompartmental analysis estimated an elimination half‐life (T_1/2_) of approximately 1–2 h, although precise estimation was limited by rapid metabolism and intra‐ and interindividual variability (Tables S1–S4). Nevertheless, this estimated half‐life range was consistent with the observed decline in plasma concentrations.

Table 3 summarizes pharmacokinetic parameters across dose groups. On Day 1, unecritinib M increased with dose, with AUC_0–t_ values of 492.0 ± 285.0 µg·h/L (200 mg), 606.0 ± 288.0 µg·h/L (250 mg), and 914.0 µg·h/L (300 mg). After repeated dosing, accumulation of metabolite was observed (Figure 1E,H). On Day 28, steady‐state AUC_0–t_ values of unecritinib M increased to 2311.0 ± 997.0 µg·h/L (200 mg), 3749.0 ± 407.0 µg·h/L (250 mg), and 3311.0 µg·h/L (300 mg). The metabolite accounted for approximately 80%–85% of total exposure for the 200 and 300 mg groups and 90% for the 250 mg group (Table 3). Accumulation ratios based on AUC (Rac_AUC), determined by dividing the AUC at Day 28 by the AUC at Day 1 (representing a single dose), were 4.7, 6.2, and 3.6 for the 200, 250, and 300 mg groups, respectively.

**TABLE 3:** Pharmacokinetics of unecritinib and unecritinib M according to dose level in pediatric patients with ALK+ ALCL.

			Dose level				
			200 mg BID (*n* = 3)		250 mg BID (*n* = 3)		300 mg BID (*n* = 1; Patient 7)
Drug	Day	Pharmacokinetic Parameter	Mean	SD	Mean	SD	Value
Unecritinib	Day 1	AUC_0‐t_, µg·h/L	89.0	67.6	71.3	37.3	235.0
		*T*_max_, h	0.8	0.3	1.2	0.8	0.5
		*C*_max_, µg/L	48.1	29.9	50.6	21.3	228.0
	Day 28	AUC_0‐t_, µg·h/L	114.0	49.3	94.8	29.1	333.0
		*T*_max_, h	0.7	0.3	1.2	0.8	2.0
		*C*_max_, µg/L	112.0	34.4	61.7	39.9	203.0
		*C*_min_, µg/L	0.2	0.3	0.2	0.3	0
		*C*_av_, µg/L	11.4	4.9	12.1	1.3	33.3
Unecritinib M	Day 1	AUC_0‐t_, µg·h/L	492.0	285.0	606.0	288.0	914.0
		*T*_max_, h	3.3	1.2	3.0	1.7	4.0
		*C*_max_, µg/L	73.4	40.8	94.3	47.0	112.0
	Day 28	AUC_0‐t_, µg·h/L	2311.0	997.0	3749.0	407.0	3311.0
		*T*_max_, h	2.3	1.5	4.0	2.0	4.0
		*C*_max_, µg/L	266.0	104.0	424.0	52.4	426.0
		*C*_min_, µg/L	174.0	82.5	280.0	7.2	205.0
		*C*_av_, µg/L	276.0	68.0	375.0	40.4	331.0

Abbreviations: ALCL, anaplastic large‐cell lymphoma; ALK+, anaplastic lymphoma kinase‐positive; AUC0‐t, area under the plasma concentration–time curve from dosing to the last measurable concentration; BID, twice daily; *C*_av_, average plasma concentration; *C*_max_, maximum plasma concentration; *C*_min_, minimum plasma concentration; M, metabolite; *n*, number of patients; SD, standard deviation; *T*_max_, time to maximum plasma concentration.

### 2.4 Treatment Responses and Clinical Outcomes

Treatment response and survival outcomes were evaluated to determine the preliminary antitumor activity and durability of unecritinib in pediatric patients with R/R ALK+ ALCL. Clinical responses were assessed according to the 2014 Lugano Classification using serial ^18^F‐labeled fluorodeoxyglucose positron emission tomography/computed tomography (CT) (^18^F‐FDG PET/CT) imaging [22].

At the data cutoff date, no patient deaths occurred, and unecritinib treatment remained ongoing in four of the nine patients (44.4%). The median number of treatment cycles administered was 31.5 (range, 3.29–56.0 cycles). Figure 2A summarizes best overall responses (BOR) and responses after two treatment cycles. The BOR rate was 100%, including six complete responses (CRs) and three partial responses (PRs). After two treatment cycles, four patients achieved CR and five achieved PR. The median time to response was 1.9 months (range, 1.8–2.0 months), and the median treatment duration was 29.4 months (range, 3.1–52.2 months) (Figure 2B). Owing to the limited cohort size and histological distribution, the association between response and histological subtype could not be evaluated.

**FIGURE 2:**
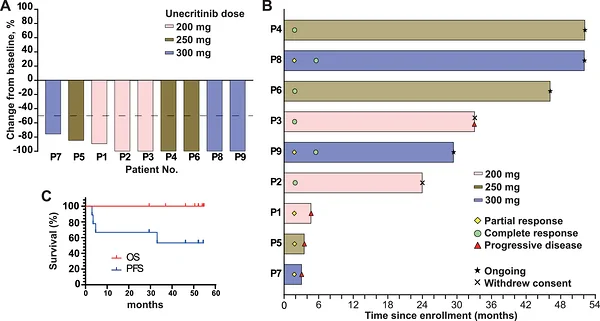
Clinical activity of unecritinib. (A) Waterfall plot showing the best percentage change in the SPD of target lesions in the nine enrolled patients. Tumor burden was centrally assessed according to the 2014 Lugano Classification using up to six measurable nodal and extranodal target lesions identified on imaging. Dashed horizontal lines at a 50% decrease indicate the threshold for PR. The ORR after two treatment cycles and the best ORR were both 100.0%, with six patients achieving CR and three achieving PR. (B) Swimmer plot showing treatment duration and clinical outcomes for each patient. The median treatment duration was 29.4 months (range, 3.1–52.2 months). Patients 2 and 3 discontinued unecritinib after withdrawal of informed consent for personal reasons. Patient 2 remained progression‐free after treatment discontinuation, whereas Patient 3 experienced relapse 3 months after treatment cessation. (C) Kaplan‒Meier curves for PFS and OS in pediatric patients with relapsed/refractory anaplastic lymphoma kinase‐ positive anaplastic large‐cell lymphoma. The estimated 3‐year PFS rate and OS rates were 53.3% and 100%, respectively. CR, complete response; No., number; ORR, objective response rate; OS, overall survival; P, patient; PFS, progression‐free survival; PR, partial response; SPD, sum of the product of perpendicular diameters for multiple lesions.

Three patients (33.3%) discontinued unecritinib because of PD after 3–4 treatment cycles. Two patients (22.2%) voluntarily withdrew treatment, and no treatment discontinuations were attributed to TRAEs. Among the two patients who voluntarily discontinued treatment, one patient experienced relapse three months after treatment cessation following 33.1 months of therapy, whereas the other remained in CR for 30.3 months after discontinuation without receiving additional antitumor therapy during follow‐up. At the data cut‐off date, the remaining four patients with CR continued unecritinib treatment for a median duration of 46.0 months (range, 29.4–52.2 months) and remained progression‐free.

All four patients with disease progression subsequently received salvage therapy, including chemotherapy, ALK inhibitors, and brentuximab vedotin (BV). At the last follow‐up, none of these patients had undergone HSCT, and all remained disease‐free.

Table 4 summarizes individual treatment courses and clinical outcomes for all nine patients. Figure 2C presents Kaplan–Meier estimates of progression‐free survival (PFS) and overall survival (OS). The 3‐year PFS rate was 53.3%, whereas the 3‐year OS rate was 100%. Median PFS and OS were not reached at the time of analysis.

**TABLE 4:** Treatments and outcomes of pediatric patients with ALK+ ALCL treated with unecritinib.

Case no.	Unecritinib dose, mg BID	Response after 2 cycles	Best overall response	PD	Outcome at last follow‐up
P1^a^	200	PR	PR	Yes^b^	NED
P2	200	CR	CR	No	NED
P3^a^	200	CR	CR	Yes^c^	NED
P4	250	CR	CR	No	NED
P5^a^	250	PR	PR	Yes^d^	NED
P6	250	CR	CR	No	NED
P7^a^	300	PR	PR	Yes^e^	NED
P8	300	PR	CR	No	NED
P9	300	PR	CR	No	NED

Abbreviations: ALK+ mediastinumALCL, anaplastic lymphoma kinase‐positive anaplastic large‐cell lymphoma; BID, twice daily; CR, complete response; NED, no evidence of disease; P, patient; PD, progressive disease; PR, partial response.

^a^ All four patients with progressive disease declined hematopoietic stem cell transplantation and remained disease‐free at the time of analysis.

^b^ After disease progression, Patient 1 received multidrug combination chemotherapy, vinblastine, and ceritinib. The patient remained on ceritinib treatment for 9 months at the time of last follow‐up.

^c^ Informed consent was withdrawn after 33.1 months of unecritinib treatment. The patient experienced relapse 3 months after treatment discontinuation and subsequently achieved CR with alectinib. Alectinib treatment was continued for 24 months.

^d^ Patient 5 was unable to afford ALK inhibitor therapy after progression. Salvage therapy included vinblastine, gemcitabine, and gemcitabine plus vinorelbine. The patient remained off treatment for 21 months at the last follow‐up.

^e^ After disease progression, Patient 7 received salvage therapy consisting of multidrug combination chemotherapy, brentuximab vedotin, vinblastine, and gemcitabine and remained on salvage treatment at the last follow‐up.

Figure 3A–D presents representative radiological responses in four patients who received unecritinib at doses of 200, 250, and 300 mg BID. All four patients had high baseline tumor burden and demonstrated rapid tumor reduction following treatment initiation. Patients 1, 5, 7, and 9 achieved PR after two treatment cycles. Patient 9 subsequently achieved CR after six cycles of unecritinib.

**FIGURE 3:**
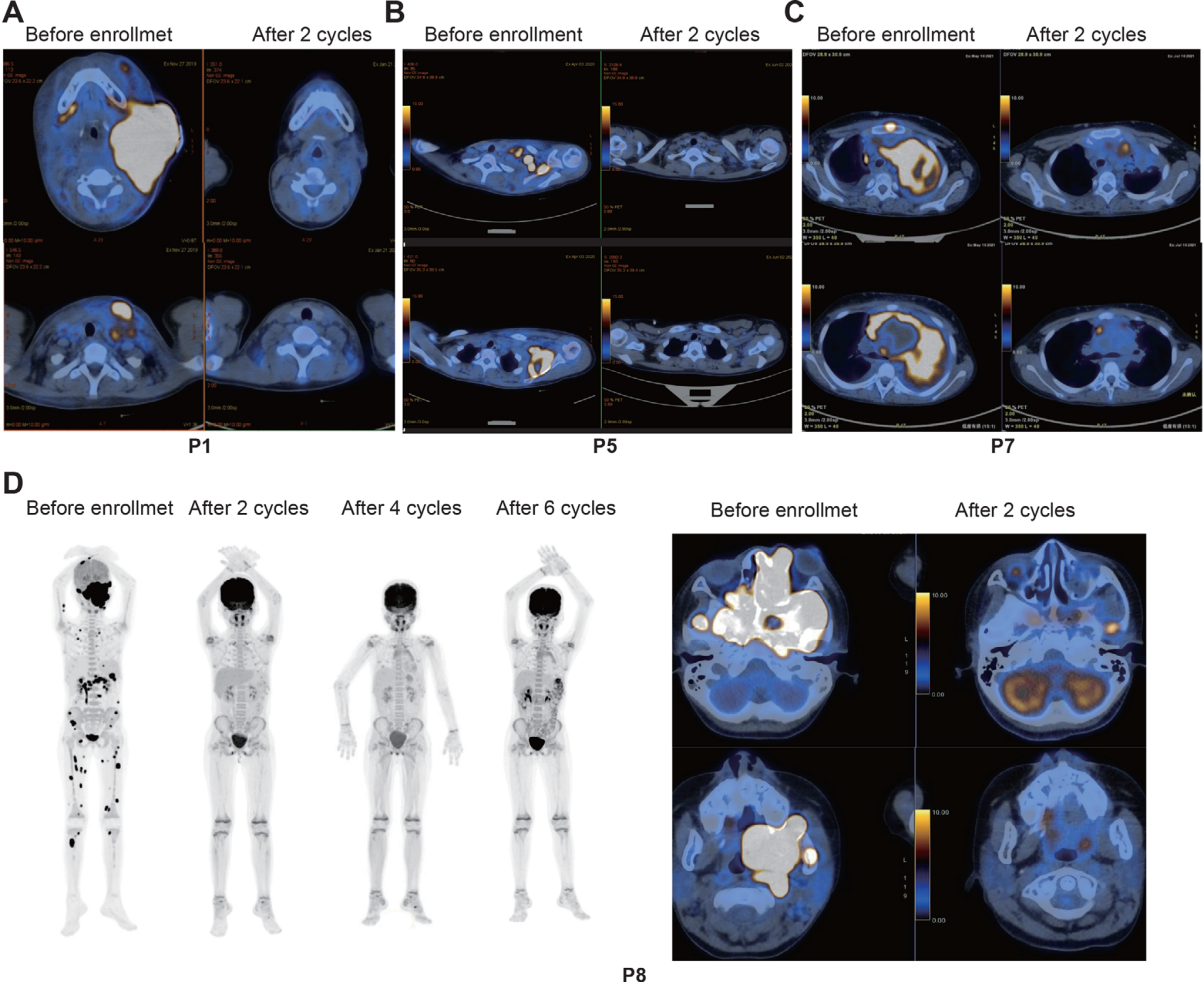
Representative radiologic responses in four patients treated with unecritinib. (A) ^18^F‐FDG‐PET/CT images of Patient 1. Before enrollment, metabolically active lesions were identified in the bilateral head and neck regions, mediastinum, axillae, left greater wing of the sphenoid bone, and left iliac bone. After two cycles of unecritinib, marked tumor regression was observed, with residual FDG‐avid lymph nodes confined to the left occipital and bilateral cervical level II–VI regions, consistent with PR. (B) ^18^F‐FDG‐PET/CT images of Patient 5. Baseline imaging demonstrated metabolically active lymphadenopathy in the left cervical IV–V, left supraclavicular, and left axillary regions, as well as lesions involving the left scapular muscle and bilateral lungs. After two treatment cycles, residual FDG uptake was limited to the left cervical level IV–V and axillary lymph nodes, consistent with PR. (C) ^18^F‐FDG‐PET/CT images of Patient 7. Before treatment, a bulky mediastinal mass invaded the left lung, bronchus, pleura, pericardium, mediastinum, and sternum, with associated pleural, pericardial, and pelvic effusions. Additional lesions involved the pleura, liver, tail of the pancreas, right lower lung, and multiple bones. After two treatment cycles, substantial tumor regression was observed, and residual mediastinal disease met criteria for PR. (D) ^18^F‐FDG‐PET/CT images of Patient 9. At recurrence, imaging revealed a skull base mass with disseminated lymph node, pulmonary, pancreatic, osseous, subcutaneous, and muscular involvement. PR was observed after two and four treatment cycles. After six cycles of unecritinib, follow‐up ^18^F‐FDG PET/CT demonstrated complete resolution of FDG‐avid disease, consistent with CR. CR, complete response; FDG, fluorodeoxyglucose; ^18^F‐FDG‐PET/CT, ^18^F‐labeled fluorodeoxyglucose positron emission tomography/computed tomography; P, patient; PET/CT, positron emission tomography/computed tomography; PR, partial response.

Overall, unecritinib demonstrated rapid and durable antitumor activity in this small cohort of pediatric patients with R/R ALK+ ALCL, with objective responses observed in all treated patients.

### 2.5 Translational Molecular Biomarker Analyses

Targeted next‐generation sequencing (NGS) was performed to characterize genomic alterations associated with ALK+ ALCL in patients treated with unecritinib. Genomic profiling was conducted using capture‐based targeted sequencing of lymphoma‐related genes in tumor‐derived DNA samples with paired blood controls.

Figure 4A–D summarizes the molecular alterations identified in six ALCL tumor samples. ALK fusions were detected in all six patients, including *nucleophosmin 1* (*NPM1*)*‐ALK* (*n* = 4), *EEF1G‐ALK* (*n* = 1), and intergenic region (*IGR*)‐ALK (*n* = 1). *MYC* alterations were identified in two patients and included one *MYC* amplification and one *ESYT2*‐*MYC* fusion.

**FIGURE 4:**
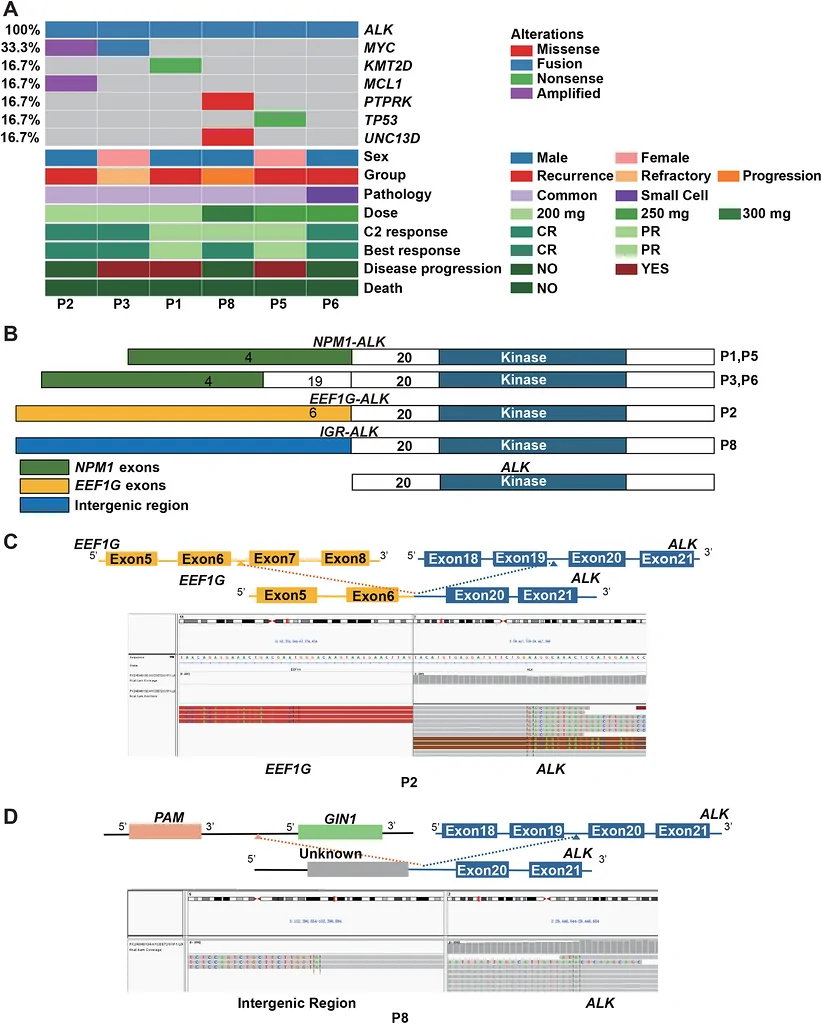
Genomic alterations identified in pediatric patients with ALK+ ALCL. (A) Heatmap showing somatic genomic alterations identified by targeted next‐generation sequencing in tumor samples from six pediatric patients with ALK+ ALCL. Detected alterations included ALK rearrangements, copy number alterations, and mutations in lymphoma‐associated genes. (B) Schematic representation of ALK fusion partner breakpoints identified in the study cohort. (C) Structural schematic of the *EEF1G‐ALK* fusion identified in Patient 2. (D) Structural schematic of the *IGR‐ALK* fusion identified in Patient 8. ALK+ ALCL, anaplastic lymphoma kinase‐positive anaplastic large‐cell lymphoma; C2, cycle 2; CR, complete response; IGR, intergenic region; P, patient; PR, partial response.

Additional genomic alterations included nonsense mutations in *KMT2D* and *TP53*, missense mutations in *PTPRK* and *UNC13D*, and *MCL1* amplification, each detected in one patient.

These findings demonstrate molecular heterogeneity among pediatric ALK+ ALCL tumors despite the universal presence of ALK rearrangements in this cohort.

## 3 Discussion

Over the past several decades, despite advances in multimodal treatment strategies for pediatric ALCL, approximately 30% of pediatric patients continue to experience relapse. More recently, ALK‐targeted therapies, including crizotinib, ceritinib, and alectinib, have demonstrated substantial clinical activity in patients with relapsed ALK+ ALCL [14, 16, 17]. To our knowledge, this is the first prospective clinical study conducted in China to evaluate unecritinib, a novel ROS1/ALK/c‐MET inhibitor, in pediatric patients with R/R ALK+ ALCL. In this Phase I study, unecritinib demonstrated a manageable safety profile and encouraging preliminary antitumor activity, with durable clinical responses observed in several patients.

Unecritinib was generally well tolerated in this pediatric cohort. No patients discontinued treatment because of treatment‐related toxicity, and no dose reductions were required. Across the evaluated dose levels (200, 250, and 300 mg BID), no clear dose‐dependent increase in toxicity was observed. Thirteen transient Grade 3 TRAEs occurred, whereas no treatment‐related Grade 4 AEs or treatment‐related deaths were reported. The overall safety profile observed in pediatric patients was generally consistent with that previously reported in adult studies of unecritinib [13] and primarily consisted of neutropenia, leukopenia, gastrointestinal AEs, and elevated AST levels. These toxicity patterns were also broadly comparable to those reported for other ALK inhibitors.

Neutropenia was the most common TRAE in the present study and occurred in 88.9% of patients. Previous pediatric studies of crizotinib reported neutropenia rates of 33% in the ALCL165 cohort (165 mg/m^2^/dose) and 70% in the ALCL280 cohort (280 mg/m^2^/dose) [14]. However, in the current study, neutropenia was transient and manageable with supportive care, and no patients required permanent treatment discontinuation or dose reduction. The most common nonhematologic TRAEs were mild gastrointestinal symptoms and transient liver function abnormalities. AEs, including neutropenia, leukopenia, nausea, and vomiting, occurred intermittently throughout treatment without evidence of cumulative toxicity.

Pulmonary toxicities and weight gain have previously been reported with several ALK inhibitors [14, 16, 17]. In the present cohort, no clinically significant pulmonary AEs or substantial treatment‐related weight gain were observed. In addition, no clinically significant growth delay or cardiac AEs were identified during long‐term follow‐up. These observations suggest that unecritinib may have a favorable long‐term tolerability profile in pediatric patients; however, larger studies with extended follow‐up are required to confirm these findings.

Ocular toxicity is a well‐recognized AE associated with ALK inhibitors. In adult patients with NSCLC, crizotinib‐associated visual impairment has been reported in up to 82% of patients [23], whereas pediatric studies have reported blurred vision and other ocular AEs during early treatment [24]. By contrast, ophthalmologic AEs in adults occurred less frequently (28.1%) [13], potentially because of lower ocular distribution of unecritinib metabolites compared with crizotinib [13]. In the present study, only three patients experienced mild ocular AEs, all of which resolved without intervention. These events included transient Grade 1 blurred vision and eye pain in Patient 9, transient Grade 1 blurred vision in Patient 5, and asymptomatic mild optic nerve injury in Patient 6 that resolved after observation. No patients required treatment interruption, dose modification, or discontinuation because of ocular toxicity.

Thromboembolic events have also been reported during crizotinib treatment, including an incidence of approximately 20% in previous studies [8]. No thromboembolic events were observed in the current cohort, although the small sample size limits definitive safety comparisons across agents.

PK analyses demonstrated that unecritinib was extensively converted to its active metabolite, unecritinib M, which accounted for most systemic drug exposure. After 28 consecutive days of twice‐daily administration, unecritinib M demonstrated substantial plasma accumulation at steady state across the evaluated dose range, consistent with previous adult PK data [13]. Exposure to unecritinib M, including AUC_0‐t_ and *C*_max_, generally increased with dose.

Currently, no established standard treatment exists for pediatric patients with R/R ALK+ ALCL. Previous preclinical and clinical studies have demonstrated that pharmacologic ALK inhibition provides meaningful clinical benefit in patients with ALK+ ALCL [14, 16, 17]. In the present study, unecritinib demonstrated encouraging efficacy and a manageable safety profile in pediatric patients with R/R ALK+ ALCL. Unecritinib achieved an objective response rate (ORR) of 100% in this cohort of nine pediatric patients, including CRs in six patients (66.7%) and PRs in three patients (33.3%). Compared with previously reported outcomes for other ALK inhibitors in similar patient populations, the observed ORR in the current study appears favorable. Specifically, crizotinib demonstrated ORRs ranging from 83% (5/6 patients in the ALCL165 cohort) to 90% (18/20 patients in the ALCL280 group) [14], whereas another study reported an ORR of 87.5% (7/8 patients) [24]. Alectinib achieved an ORR of 80% (8/10 patients), including six CRs [17], whereas ceritinib achieved an ORR of 75% (6/8 patients) [16]. Notably, responses to unecritinib were observed across all evaluated dose levels (200–300 mg BID), suggesting antitumor activity throughout the tested dosing range.

Regarding long‐term efficacy, direct comparisons of PFS across studies should be interpreted cautiously because of differences in study design and patient characteristics, follow‐up duration, and treatment settings. Nevertheless, the prolonged responses observed in the present cohort compare favorably with available data for other ALK inhibitors. Unecritinib achieved 1‐year and 3‐year PFS rates of 66.7% and 53.3%, respectively; furthermore, six patients maintained responses for > 24 months (range, 24.0–52.2 months) without HSCT consolidation. In previously reported studies, ceritinib demonstrated an estimated 1‐year PFS rate of 67% in patients with ALCL [16], although durable disease control beyond 1 year was not clearly demonstrated. Similarly, the reported 1‐year PFS rate for alectinib was 58.3% [17]. In frontline treatment settings, crizotinib combined with chemotherapy improved event‐free survival (EFS) [8] but was associated with a 20% incidence of thromboembolic events.

In addition to its clinical activity and safety profile, unecritinib may address an important unmet need in China as a domestically developed ALK inhibitor. Compared with imported ALK inhibitors, including crizotinib, alectinib, and ceritinib, domestically developed targeted therapies may improve treatment accessibility and reduce financial burden for pediatric patients requiring prolonged treatment. This potential accessibility, together with the observed efficacy and tolerability profile, supports further investigation of unecritinib in pediatric patients with R/R ALK+ ALCL.

Several salvage therapies have demonstrated activity in pediatric patients with R/R ALK+ ALCL, particularly vinblastine and BV. Weekly vinblastine achieved an ORR of 83% (25/30 evaluable patients), with multiple patients achieving CR [25]. However, long‐term outcomes remain limited, with reported 5‐year EFS and OS rates of 30% and 65%, respectively [25]. Similarly, BV achieved an ORR of 81% in patients with R/R ALCL [26]. In that study, 16 of the 38 patients who achieved CR subsequently underwent consolidative stem cell transplantation, yet the estimated 5‐year PFS and 5‐year OS rates remained 39% and 60%, respectively [26].

In the present study, unecritinib demonstrated encouraging preliminary efficacy, even without HSCT consolidation. The observed 100% ORR and 100% 3‐year OS rate, together with a 53.3% 3‐year PFS rate, compare favorably with previously reported historical outcomes for vinblastine and BV in the salvage setting. However, these findings should be interpreted cautiously because of the small sample size, single‐arm study design, and shorter follow‐up duration relative to historical comparator studies. Larger prospective studies with extended follow‐up are necessary to further define the durability of response and long‐term survival outcomes associated with unecritinib treatment.

HSCT remains an important consolidation strategy for selected patients with R/R ALK+ ALCL [27] and may provide long‐term disease control in both autologous [28, 29, 30] and allogeneic transplantation settings [28, 31, 32, 33]. Reported 5‐year EFS rates ranged from 0% to 59% after salvage therapy followed by autologous HSCT [28, 29, 30] and from 33% to 75% after allogeneic HSCT [28, 31, 32, 33]. In the current study, all enrolled patients declined HSCT. Previous studies of crizotinib reported prolonged treatment durations in several patients receiving ALCL165 (58.88, 65.75, and 71.24 months) and ALCL280 (39.88 months) dosing regimens [14]. Similarly, in the present study, six patients remained on unecritinib treatment for more than 24 months (24.0, 29.4, 33.1, 46.2, 52.1, and 52.2 months).

Long‐term outcomes among patients who achieved CR may provide preliminary insight into the durability of remission with unecritinib. One patient experienced relapse 3 months after treatment discontinuation, whereas another patient maintained CR for 30.3 months after discontinuing unecritinib without receiving additional antitumor therapy. These observations suggest that prolonged disease control may be achievable in some pediatric patients without HSCT consolidation. However, because only a limited number of patients discontinued treatment, the optimal duration of unecritinib therapy remains unclear. The relapse observed in the patient harboring *MYC* fusion may also suggest potential biologic heterogeneity within this disease population, although no definitive conclusions can be drawn from the current sample size. Additional studies are required to clarify the role of molecular alterations in treatment response and to determine optimal strategies for treatment duration and discontinuation.

In ALK+ ALCL, more than 80% of cases harbor the classic t(2;5)(p23;q35) translocation involving the *ALK* and *NPM1* genes [34]. The remaining ALK+ ALCL cases contain variant ALK fusion partners other than *NPM1*. The *EEF1G‐ALK* rearrangement is extremely rare, with only two previously reported cases in the literature [35, 36]. Consistent with a previous report [36], Patient 2 in the current study harbored breakpoints involving the same exon sites within *EEF1G* (exon 6) and *ALK* (exon 20). Previous functional studies demonstrated that *EEF1G‐ALK* fusion retains dimerization capability, constitutively activates ALK kinase signaling, and exhibits oncogenic properties similar to those of *NPM1‐ALK* [36]. In the present study, Patient 2 experienced disease relapse before enrolment but subsequently achieved CR following unecritinib treatment. In addition, *IGR‐ALK* rearrangement was identified in Patient 8. To our knowledge, this rearrangement has not been previously reported and requires further functional characterization to demonstrate its biologic significance.

Although ALK+ ALCL is generally associated with favorable outcomes, chemoresistant and clinically aggressive disease subsets have been described. In the present study, all patients initially responded to unecritinib treatment; however, three patients with high tumor burden at enrolment subsequently experienced disease progression. Sequencing data were available for two of these patients, both of whom harbored *NPM1‐ALK* rearrangements in addition to secondary genomic alterations, including *KMT2D* mutation in one patient and *TP53* mutation in another. *TP53* mutations have previously been associated with adverse prognosis in ALCL [37], whereas *KMT2D* has also been proposed as a potential prognostic contributor in this disease [37]. *MYC* alterations were additionally detected in the current cohort. Concurrent ALCL and *MYC* rearrangements are rare in ALCL and have previously been associated with aggressive clinical behavior [38]. These findings suggest that disease progression in some patients with ALK+ ALCL may be influenced not only by *ALK* fusion status itself but also by co‐occurring genomic alterations in tumor suppressor genes and oncogenic driver genes.

Despite the encouraging antitumor activity observed with unecritinib, resistance to *ALK* inhibition remains an important clinical challenge. Similar to other ALK inhibitors, including lorlatinib [39], resistance mechanisms may involve both on‐target and off‐target molecular alterations. However, because of the limited sample size and challenges associated with obtaining longitudinal tumor samples in pediatric patients with R/R ALCL, the present study was unable to comprehensively characterize mechanisms of acquired resistance. Further translational investigations with larger cohorts and serial molecular profiling are warranted to better define resistance pathways associated with unecritinib treatment.

In summary, the present preliminary findings suggest that unecritinib demonstrates clinically meaningful antitumor activity in pediatric patients with R/R ALK+ ALCL. Responses were durable in several patients, and treatment was generally well tolerated. Larger prospective studies with extended follow‐up are warranted to evaluate the efficacy, long‐term safety, and optimal clinical application of unecritinib in this patient population.

## 4 Materials and Methods

### 4.1 Study Design and Treatment

This was an open‐label, single‐arm, single‐center, Phase I clinical trial conducted at Sun Yat‐sen University Cancer Center (SYSUCC) using a standard 3 + 3 dose‐escalation design. In a previous Phase I study of unecritinib in adult patients with *ROS1*‐positive advanced NSCLC, patients received unecritinib at doses of 100, 200, and 300 mg once daily and 200, 250, 300, and 350 mg BID following a 3 + 3 dose‐escalation design [13]. That study identified 350 mg BID as the MTD and 300 mg BID as the RP2D [13]. Based on the findings of the adult Phase I study [13], the starting dose of unecritinib in pediatric patients aged 10–18 years was 200 mg BID, corresponding to approximately 80% of the adult MTD. The dose levels evaluated in this study were 200, 250, and 300 mg BID. Each treatment cycle was 28 days in duration.

The primary endpoints were safety, RP2D determination, and PK. Secondary endpoints included ORR, PFS, and OS. Unecritinib treatment was discontinued in patients who developed PD. Additional criteria for study discontinuation included disease progression, withdrawal of informed consent, unacceptable toxicity, or investigator decision. Patients who achieved CR were permitted to undergo HSCT.

### 4.2 Patients

Patients with R/R ALK+ ALCL were enrolled. ALK status was centrally assessed at SYSUCC, which served as the sponsor‐designated central laboratory.

The inclusion criteria were as follows: (a) age 10–18 years; (b) Eastern Cooperative Oncology Group performance status of 0 or 1; (c) presence of an ALK fusion confirmed by immunohistochemistry, Clinical Laboratory Improvement Amendments (CLIA)‐certified fluorescence in situ hybridization, reverse transcription‐polymerase chain reaction, or NGS; (d) R/R ALK+ ALCL; (e) at least one measurable lesion according to the 2014 Lugano Classification [22], primarily assessed using ^18^F‐FDG PET/CT; (f) estimated life expectancy ≥ 3 months; (g) adequate organ function, defined as absolute neutrophil count (ANC) ≥ 1500/mm^3^ (or ≥ 1000/mm^3^ in patients with known bone marrow involvement), platelet count ≥ 75,000/mm^3^ (or ≥ 50,000/mm^3^ in patients with known bone marrow involvement), bilirubin ≤ 1.5 × upper limit of normal (ULN), serum creatinine ≤ 1.5 × ULN, ALT and AST ≤ 3 × ULN; and (h) written informed consent obtained from all patients and their guardians before study initiation.

Patients were excluded if they (a) had primary cutaneous ALCL, (b) had previously received treatment with an ALK inhibitor, or (c) had previously undergone allogeneic HSCT.

### 4.3 DLT

The DLT evaluation period comprised the first treatment cycle. DLT was defined as any event considered definitely, probably, or possibly related to unecritinib, including but not limited to the following: (a) Grade 4 hematologic toxicity, including Grade 4 thrombocytopenia, Grade 4 neutropenia, or febrile neutropenia; (b) any Grade ≥ 3 nonhematologic toxicity, excluding Grade 3 alopecia or electrolyte abnormalities resolving within 3 days; or (c) Grade ≥ 3 nausea, vomiting, or diarrhea despite appropriate symptomatic treatment.

The MTD was defined as the highest dose level at which fewer than one‐third of patients experienced DLT during Cycle 1. The RP2D was established at or below the MTD.

### 4.4 Safety Evaluation

Toxicities were graded according to the National Cancer Institute Common Terminology Criteria for Adverse Events, version 5.0. The relative frequency of each TRAE considered likely, probably, or possibly related to unecritinib was calculated as the proportion of toxicity‐evaluable cycles in which the corresponding TRAE occurred. AEs were recorded from the first day of treatment through at least 28 days after the final dose. Comprehensive ophthalmologic evaluations, including visual acuity assessment and slit‐lamp examination, were performed during each treatment cycle.

### 4.5 PK Assessments

Blood samples for PK analysis were collected before unecritinib administration on Days 1, 7, 14, 21, and 28 and at 0.5, 1, 2, 4, 6, and 10 h after dosing on Days 1 and 28. Samples were immediately centrifuged, and plasma was isolated and stored at −80°C until analysis. Plasma concentrations of unecritinib and unecritinib M were quantified using high‐performance liquid chromatography–tandem mass spectrometry.

PK parameters for unecritinib and unecritinib M were derived using noncompartmental analysis with WinNonlin software version 8.2 (Certara, Princeton, NJ, USA). Area‐under‐the‐curve calculations were performed using the linear‐log trapezoidal method. PK parameters included *C*_max_, *T*_max_, minimum concentration (*C*_min_), area under the concentration‐time curve from dosing to the last measurable concentration (AUC_0‐t_), average concentration (*C*_av_), and fluctuation ratio. PK results were summarized by cohort and graphically presented using GraphPad Prism version 10 (San Diego, CA, USA).

### 4.6 Response Assessment

Treatment response was assessed according to the 2014 Lugano Classification, which primarily relies on ^18^F‐FDG PET/CT [22]. Responses in FDG‐avid disease were evaluated using a 5‐point scale. After study enrollment, ^18^F‐FDG PET/CT scans were performed every 2 cycles through 8 cycles, and every 4 cycles thereafter. Minimal disseminated disease testing was optionally performed to further characterize treatment response.

### 4.7 Targeted Gene Panel Sequencing

Genetic profiling was performed using capture‐based targeted NGS. Genomic DNA was extracted from liquid biopsy samples collected at enrollment. Genomic DNA isolated from matched white blood cells was used as the normal control for germline variant filtering. Targeted NGS of 475 lymphoma‐related genes, including all exons and fusion‐associated intronic regions of ALK, was performed at a centralized CLIA‐certified and College of American Pathologists‐accredited clinical testing laboratory (Nanjing Geneseeq Technology Inc., Nanjing, China) according to protocols approved by the Ethics Committee of SYSUCC.

The mean sequencing coverage depth was 100× for controls, 1000× for formalin‐fixed paraffin‐embedded samples, and approximately 5000× for circulating cell‐free DNA samples. Somatic genomic alterations, including single‐nucleotide variants, insertions/deletions, and copy number variants, were analyzed as previously described [40]. Structural variants were identified using FACTERA with default parameters. Fusion‐supporting reads were manually reviewed and confirmed using Integrative Genomics Viewer.

### 4.8 Statistical Analysis

The sample size for this Phase I trial was determined using a standard 3 + 3 design. Statistical analyses were performed using GraphPad Prism software version 10.0. Categorical variables were summarized as frequencies and percentages, whereas continuous variables were summarized as ranges.

PFS was defined as the interval from study enrollment to disease progression, death from any cause, or last follow‐up in patients without an event. OS was defined as the interval from enrollment to death from any cause or last follow‐up. PFS and OS were estimated using the Kaplan–Meier method.

## Author Contributions

Z.Y.Z. and H.Q.H. conceived and designed the experiments. S.Y.L. wrote the manuscript. Z.Y.Z., J.T.H. and F.F.S. revised the manuscript. S.Y.L., J.T.H., R.Q.C., Y.G., J.Z., J.W., Y.Q., Z.J.Z., and F.F.S. contributed to data acquisition, including patient enrollment and treatment. S.Y.L., J.T.H., R.Q.C., and F.F.S. performed the data analysis and interpretation of the manuscript. All authors have reviewed and approved the manuscript.

## Funding

This study was supported by the National Key Research and Development Program of China (no. 2022YFC2705005) and the National Natural Science Foundation of China (nos. 82573714, 82203303, and 82302940). This study was also supported by Chia Tai Tianqing Pharmaceutical Group Co., Ltd., Nanjing, China, which did not participate in the study design, data collection, analysis, interpretation, or writing of the manuscript.

## Ethics Statement

This trial was approved by the Institutional Review Board and Ethics Committee of SYSUCC (approval no. A2019‐019‐01). The study was registered at ClinicalTrials.gov (NCT04306887). Written informed consent was obtained from the legal guardians of all pediatric subjects. Furthermore, in accordance with pediatric ethical guidelines, written assent was additionally obtained from children with the cognitive capacity to understand the trial. We strictly respected the minors' right to refuse or withdraw from the study at any time, which would prevail over guardians' consent, except in life‐threatening situations where the investigator and guardian determined that trial treatment was essential for the patient's survival. In addition, if any minor participant reached the legal age of consent during the trial, they were required to sign their own informed consent form before continuing trial procedures. Animal experiments: Not applicable; this was a Phase I clinical trial that did not involve animal research.

## Conflicts of Interest

The authors declare the following potential competing interests: Nanjing Geneseeq Technology Inc. provided targeted NGS services and technical support for the bioinformatics analysis of the genomic data. Chia Tai Tianqing Pharmaceutical Group Co., Ltd. provided financial support for this study but had no role in the study design, data collection, analysis, interpretation, or writing of the manuscript. All authors declare no other conflicts of interest.

## Supporting information




**Supporting File 1**: Mco271128‐sup‐0001‐SuppMat.docx

## Data Availability

Translational Genomic Data: The raw sequence data reported in this paper have been deposited in the Genome Sequence Archive (Genomics, Proteomics & Bioinformatics 2025) in National Genomics Data Center (Nucleic Acids Res 2025), China National Center for Bioinformation / Beijing Institute of Genomics, Chinese Academy of Sciences (GSA‐Human: HRA018012) that are publicly accessible at https://ngdc.cncb.ac.cn/gsa‐human. Clinical Research Data: The core clinical trial data have been officially registered and backed up in the Research Data Deposit (RDD) platform under the tracking identifier RDDA2026693604 (https://www.researchdata.org.cn/). Additional data supporting the findings of this study are available from the corresponding authors upon reasonable request and with permission from Sun Yat‐sen University Cancer Center.
