# Supplementary material for: Safety, Efficacy, and Pharmacokinetics of Unecritinib (TQ‐B3101) in Pediatric Patients With Relapsed/Refractory Anaplastic Lymphoma Kinase‐Positive Anaplastic Large‐Cell Lymphoma: A Phase I Study
Source: MedComm (2020). 2026 Nov 5;7(11):e71128. doi: 10.1002/mco2.71128 (PMC13740676; doi:10.1002/mco2.71128)
Supplement: Supplementary file 1 [file MCO2-7-e71128-s001.docx]

Table S1. Pharmacokinetic data after a single dose of unecritinib.

| **Dose (mg)** | **Subject** | **Half-life (T_1/2_)** | **C_max_, μg/L** | **AUC_0-t_, μg·h/L** | **T_max_, h** |
| --- | --- | --- | --- | --- | --- |
| 200 | P1 | 0.9 | 65.0 | 76.4 | 0.5 |
|  | P2 |  | 13.6 | 28.5 | 1.0 |
|  | P3 | 0.8 | 65.7 | 162.0 | 1.0 |
|  | Mean | 0.9 | 48.1 | 89.0 | 0.8 |
|  | SD | 0.1 | 29.9 | 67.6 | 0.3 |
| 250 | P4 | 2.3 | 74.7 | 98.8 | 1.0 |
|  | P5 |  | 34.2 | 86.3 | 2.0 |
|  | P6 |  | 43.0 | 28.9 | 0.5 |
|  | Mean | 2.3 | 50.6 | 71.3 | 1.2 |
|  | SD |  | 21.3 | 37.3 | 0.8 |
| 300 | P7 | 1.5 | 228.0 | 235.0 | 0.5 |
|  | Mean | 1.5 | 228.0 | 235.0 | 0.5 |
|  | SD |  |  |  |  |

Abbreviations: AUC_0-t_, area under the plasma concentration–time curve from dosing to the last measurable concentration; C_max_, maximum plasma concentration; h, hour; P, patient; SD, standard deviation; T_1/2_, half-life; T_max_, time to maximum plasma concentration.

Table S2. Pharmacokinetic data after multiple doses of unecritinib.

| **Dose (mg)** | **Subject** | **Half-life (T_1/2_)** | **Fluctuation ratio (%)** | **C_max_, μg/L** | **AUC_0-t_, μg·h/L** | **T_max_, h** |
| --- | --- | --- | --- | --- | --- | --- |
| 200 | P1 | 2.5 | 855.0 | 81.5 | 95.4 | 0.5 |
|  | P2 | 1.4 | 1340.0 | 104.0 | 76.9 | 0.5 |
|  | P3 | 2.3 | 872.0 | 149.0 | 170.0 | 1.0 |
|  | Mean | 2.1 | 1022.3 | 112.0 | 114.0 | 0.7 |
|  | SD | 0.6 | 275.2 | 34.4 | 49.3 | 0.3 |
| 250 | P4 |  | NA | 28.8 | 63.5 | 2.0 |
|  | P5 | 2.2 | 413.0 | 50.2 | 121.0 | 1.0 |
|  | P6 | 1.1 | 1040.0 | 106.0 | 100.0 | 0.5 |
|  | Mean | 1.7 | 726.5 | 61.7 | 94.8 | 1.2 |
|  | SD | 0.7 | 443.4 | 39.9 | 29.1 | 0.8 |
| 300 | P7 | 2.0 | 609.0 | 203.0 | 333.0 | 2.0 |
|  | Mean | 2.0 | 609.0 | 203.0 | 333.0 | 2.0 |
|  | SD |  |  |  |  |  |

Abbreviations: AUC_0-t_, area under the plasma concentration–time curve from dosing to the last measurable concentration; C_max_, maximum plasma concentration; h, hour; P, patient; SD, standard deviation; T_1/2_, half-life; T_max_, time to maximum plasma concentration.

Table S3. Pharmacokinetic data after a single dose of unecritinib M.

| **Dose (mg)** | **Subject** | **Half-life (T_1/2_)** | **C_max_, μg/L** | **AUC_0-t_, μg·h/L** | **T_max_, h** |  |
| --- | --- | --- | --- | --- | --- | --- |
| 200 | P1 |  | 30.0 | 192.0 | 4.0 |  |
|  | P2 |  | 79.3 | 526.0 | 4.0 |  |
|  | P3 | 5.5 | 111.0 | 758.0 | 2.0 |  |
|  | Mean | 5.5 | 73.4 | 492.0 | 3.3 |  |
|  | SD |  | 40.8 | 285.0 | 1.2 |  |
| 250 | P4 |  | 108.0 | 789.0 | 4.0 |  |
|  | P5 |  | 133.0 | 755.0 | 4.0 |  |
|  | P6 | 4.9 | 42.0 | 274.0 | 1.0 |  |
|  | Mean | 4.9 | 94.3 | 606.0 | 3.0 |  |
|  | SD |  | 47.0 | 288.0 | 1.7 |  |
| 300 | P7 |  | 112.0 | 914.0 | 4.0 |  |
|  | Mean |  | 112.0 | 914.0 | 4.0 |  |
|  | SD |  |  |  |  |  |

Abbreviations: AUC_0-t_, area under the plasma concentration–time curve from dosing to the last measurable concentration; C_max_, maximum plasma concentration; M, metabolite; h, hour; P, patient; SD, standard deviation; T_1/2_, half-life; T_max_, time to maximum plasma concentration.

Table S4. Pharmacokinetic data after multiple doses of unecritinib M.

| **Dose (mg)** | **Subject** | **Half-life (T_1/2_)** | **Fluctuation ratio (%)** | **C_max_, μg/L** | **AUC_0-t_, μg·h/L** | **T_max_, h** |  |
| --- | --- | --- | --- | --- | --- | --- | --- |
| 200 | P1 |  | 41.0 | 150.0 | 1294.0 | 4.0 |  |
|  | P2 | 38.6 | 27.1 | 350.0 | 3286.0 | 1.0 |  |
|  | P3 | 16.7 | 57.0 | 297.0 | 2352.0 | 2.0 |  |
|  | Mean | 27.6 | 43.1 | 266.0 | 2311.0 | 2.3 |  |
|  | SD | 15.5 | 22.1 | 104.0 | 997.0 | 1.5 |  |
| 250 | P4 |  | 38.1 | 435.0 | 3910.0 | 6.0 |  |
|  | P5 | 16.7 | 48.9 | 470.0 | 4050.0 | 2.0 |  |
|  | P6 |  | 25.9 | 367.0 | 3286.0 | 4.0 |  |
|  | Mean | 16.7 | 37.6 | 424.0 | 3749.0 | 4.0 |  |
|  | SD |  | 11.5 | 52.4 | 407.0 | 2.0 |  |
| 300 | P7 |  | 66.8 | 426.0 | 3311.0 | 4.0 |  |
|  | Mean |  | 66.8 | 426.0 | 3311.0 | 4.0 |  |
|  | SD |  |  |  |  |  |  |

Abbreviations: AUC_0-t_, area under the plasma concentration–time curve from dosing to the last measurable concentration; C_max_, maximum plasma concentration; M, metabolite; h, hour; P, patient; SD, standard deviation; T_1/2_, half-life; T_max_, time to maximum plasma concentration.
